# Interactions of anthropometric indices, rs9939609 FTO gene polymorphism and breast cancer: A case‐control study

**DOI:** 10.1111/jcmm.16394

**Published:** 2021-02-25

**Authors:** Saeid Doaei, Fatemeh Bourbour, Samira Rastgoo, Mohammad Esmail Akbari, Maryam Gholamalizadeh, Azadeh Hajipour, Alireza Moslem, Fereshteh Ghorat, Mostafa Badeli, Seyedeh Elaheh Bagheri, Atieh Alizadeh, Zohreh Mokhtari, Samaneh Pishdad, Sepehr JavadiKooshesh, Ghasem Azizi Tabesh, Fateme Montazeri, Parvin Joola, Shahla Rezaei, Masoomeh Dorosti, Seyed Alireza Mosavi Jarrahi

**Affiliations:** ^1^ Cancer Research Center Shahid Beheshti University of Medical Sciences Tehran Iran; ^2^ Department of Clinical Nutrition and Dietetics Shahid Beheshti University of Medical Science Tehran Iran; ^3^ Student Research Committee Cancer Research Center, Shahid Beheshti University of Medical Sciences Tehran Iran; ^4^ Department of Health Sciences in Nutrition, School of Health Qazvin University of Medical Sciences Qazvin Iran; ^5^ Iranian Research Center on Healthy Aging Sabzevar University of Medical Sciences Sabzevar Iran; ^6^ Non‐Communicable Diseases Research Center Sabzevar University of Medical Sciences Sabzevar Iran; ^7^ Department of Nutrition Urmia University of Medical Science Urmia Iran; ^8^ School of Paramedicine Guilan University of Medical Sciences Langroud Iran; ^9^ Department of Clinical Biochemistry, Faculty of Medicine Shahid Beheshti University of Medical Sciences Tehran Iran; ^10^ Nutrition and Food Security Research Center Shahid Sadoughi University of Medical Sciences Yazd Iran; ^11^ Department of Medical Genetics, School of Medicine Shahid Beheshti University of Medical Sciences Tehran Iran; ^12^ Genomic Research center Shahid Beheshti University of Medical Sciences Tehran Iran; ^13^ Department of non‐communicable diseases, deputy of health services Dezful University of Medical Sciences Dezful Iran; ^14^ Student Research Committee, Department of Clinical Nutrition, School of Nutrition and Food Sciences Shiraz University of Medical Sciences Shiraz Iran

**Keywords:** anthropometric indices, breast cancer, fat mass‐ and obesity‐associated, polymorphism

## Abstract

Contradictory results were reported on the effect of fat mass‐ and obesity‐associated (FTO) gene and anthropometric measurements on breast cancer (BC). This study aimed to assess the interactions between rs9939609 polymorphism of FTO gene, anthropometric indices and BC risk in Iranian women. This case‐control study was performed on 540 women including 180 women with BC and 360 healthy women in Tehran, Iran. Physical activity and dietary intakes were assessed by validated questionnaires. Data on sociodemographic and pathologic factors of the participants as well as their blood samples were collected. The rs9939609 FTO gene polymorphism was genotyped using the tetra‐primer amplification refractory mutation system‐polymerase chain reaction (T‐ARMS‐PCR). No significant association was found between BC and risk allele of FTO rs9939609 polymorphism after adjustments for the confounders. However, there was a significant association between rs9939609 polymorphism risk allele and BC risk in females with overweight, even after adjusting for age, family history of BC, abortion, BMI and the number of pregnancies (*P* < .05). The association was disappeared after further adjustments for lifestyle factors including smoking, alcohol consumption, calorie and macronutrients intake, and physical activity. The FTO gene polymorphism was associated with the risk of BC in overweight individuals. This association was influenced by environmental factors including diet, alcohol consumption and smoking. Future studies are required to confirm the association between the FTO gene and BC in overweight females and to identify the underlying mechanisms.

## 1 INTRODUCTION

Breast cancer (BC) is the most frequently diagnosed cancer with an incidence rate of 10.4% of all cancers and the most common cause of cancer death in women worldwide. It is a major public health problem, which is responsible for 1 million of about 10 million new neoplasms which are diagnosed every year around the world.^1^, ^2^, ^3^, ^4^ The prevalence of BC is increasing which may be partly due to lifestyle issues and the obesity pandemic.^3^, ^5^ Early menstruation, late menopause, having the first child at an older age, smoking, hormonal replacement therapy, contraceptive medications and lack of breastfeeding have all been identified as the risk factors for developing BC.^6^, ^7^ Furthermore, dietary intake,^5^ obesity and anthropometric indices are the other important factors associated with BC.^8^ Obesity has been consistently associated with an increased risk of postmenopausal breast cancer in population‐based studies. Conversely, obesity in such studies has been inversely associated with premenopausal breast cancer risk.^9^

Fat mass‐ and obesity‐associated (FTO) gene have been reported to play an important role in the aetiology of both obesity and BC.^9^, ^10^ FTO gene is expressed in different human tissues^11^ and plays a role in the regulation of cell metabolism.^12^ The expression of FTO is reported to be regulated by dietary intake and nutritional status.^13^, ^14^ Many studies have also reported a significant association between FTO genotype and anthropometric measurements such as body mass index (BMI), body weight and body composition.^15^ For example, the carriers of the A allele of FTO rs9939609 polymorphism have a higher body fat percentage.^16^, ^17^, ^18^ The homozygotes for the FTO rs9939609 risk allele (A) had higher serum leptin,^16^ and the amounts of dietary calorie, carbohydrate and fat intake were associated with FTO genotype.^17^ The obesity‐related SNPs reside in the first intron of FTO, and they may not only impact FTO but mediate their obesity effects via nearby genes such as IRX3.^18^

On the other hand, recent studies have reported that there is a strong association between FTO single nucleotide polymorphisms (SNPs) such as rs9939609 with the increased risk of some types of BC, implying a possible mediatory role of FTO in the pathogenesis of cancers.^10^, ^19^ The FTO may act as an amino acid sensor, linking circulating amino acid to the mammalian target of rapamycin complex 1 (mTORc1). This may be fundamental to its role in cell growth and proliferation.^18^ However, some other studies have failed to find any association between FTO gene rs9939609 polymorphism and BC.^20^, ^21^, ^22^, ^23^ These contradictory results may be due to the effects of the other related factors on this association. Moreover, the association between anthropometric indices and cancer has been frequently reported.^8^, ^24^ However, the effect of anthropometric indices on the manifestation of the effect of the FTO risk allele on BC is not yet clear. Based on the previous studies,^9^, ^16^ body weight and BMI may influence the effect of FTO gene on the risk of BC.

Given the high prevalence of BC and inconclusive results of studies, this case‐control study was aimed to identify the interactions between anthropometric indices, FTO gene rs9939609 polymorphism and BC risk in Iranian women.

## 2 METHODS

### 2.1 Study population and data collection

A case‐control study was performed on 540 adult women including 180 patients with cancer as the case group and 360 healthy individuals as the control group. A 1:2 case‐to‐control ratio was used in this matched case‐control study due to concern for sufficient numbers in stratified analysis and increase in power given the expected prevalence of exposure among the controls. The required sample size was estimated according to a previous similar study.^10^ The cases were selected according to the inclusion criteria from adult women referring to the Cancer Research Center of Shohada Tajrish Hospital in Tehran, Iran. The controls were selected from the adult women who participate in Sabzevar Persian cohort study. The inclusion criteria for the case group included females with BC, age between 35 and 70 years, consent to participate in the study, lack of diseases affecting body weight, do not take drugs that affect body weight and not more than 3 months after the BC diagnosis. The inclusion criteria of the control group included females with no malignancy, age between 35 and 70 years, lack of diseases affecting body weight, do not take drugs that affect body weight and consent to participate in the study. Basic information including medical history, physical activity using the International Physical Activity Questionnaire (IPAQ), alcohol consumption and smoking, the level of education, socio‐economic factors and food intake using a validated Food Frequency Questionnaire (FFQ) was collected.

### 2.2 Genotyping

Five millimetres (ml) of the blood samples of the participants was collected at the beginning of the study. The extracted deoxyribonucleic acid (DNA) samples were amplified using polymerase chain reaction (PCR) and master mix DNA polymerase (cat. No A180301; Ampliqon). The PCR products were examined to identify rs9939609 polymorphism of the FTO gene by the Tetra‐primer amplification refractory mutation system (ARMS)‐PCR method.

### 2.3 Anthropometric indicators

Height was measured using a stadiometer. The patient's weight was measured using a SECA Alpha 882 scale (SECA Corporation). The patients' BMI was then calculated by dividing the weight by the square of height.

### 2.4 Statistical analysis

The two groups were compared in terms of demographic, anthropometric, clinical indicators and the presence of FTO gene polymorphism at the beginning of the study using t test (for quantitative variables) and chi‐square (for qualitative variables) methods. Then, the relationship between BC and the risk allele of rs9939609 polymorphism (the dominant genetic model) was investigated using the logistic regression method.

The effects of confounding variables were adjusted using different models in the logistic regression method including age, family history of BC, menopausal status, lactation time, history of abortion, age of onset of menstruation, number of pregnancies and BMI model 1, and further adjustments for smoking, alcohol consumption, calorie intake, macronutrients intake and physical activity in Model 2. In the next step, to investigate the effect of BMI on the relationship between FTO gene polymorphism and BC risk, statistical analysis was limited to overweight people (BMI > 27). All statistical analyses were performed using SPSS software ver. 21.0 (IBM) and considering the significance level of *P* < .05.

## 3 RESULTS

The mean BMI of the cases and controls was 29 (±2.8) and 27 (±2.3), respectively (*P* < .01). In the case group, the rate of breastfeeding months and the number of pregnancies were significantly lower than the control group (both *P* = .01), while the family history of BC, calorie intake and carbohydrate intake in the case group were significantly higher than the control group (all *P* = .01, Table 1).

**TABLE 1:** Characteristics of the participants

	Cases (170)	Controls (360)	*P*
Age (y)	68 (±29)	65 (±27)	.06
Height (cm)	156 (±5)	161 (±6)	.01
Weight (kg)	71 (±11)	71 (±10)	.86
BMI (kg/m^2^)	29.19 (±2.8)	27.27 (±2.3)	.01
Breastfeeding duration (mo)	34 (±29)	59 (±33)	.01
First menstruation age (y)	13 (±2)	13 (±2)	.51
Menopausal age (y)	47 (±5)	47 (±5)	.89
Menopausal women	110 (65%)	223 (62%)	.66
Family history of BC	60 (35%)	50 (14%)	.01
Number of pregnancies	3 (±2)	4 (±2)	.01
Smoking	5 (2.9%)	18 (5%)	.34
Using alcohol drinks	168 (98.5%)	357 (99.2%)	.62
Physical activity (h/d)	2 (±4.5)	1.5 (±1.5)	.51
FTO genotype for rs9939609 polymorphism			
TT	22 (31%)	126 (35%)	.79
AT	19 (11%)	32 (9%)	
AA	99 (58%)	202 (56%)	
Dominant model			
TT	53 (31%)	126 (35%)	.46
AA + AT	117 (69%)	234 (65%)	
Calorie intake (Kcal)	2737 (±925)	2315 (±106)	.01
Protein intake (g/d)	87 (±42)	85 (±42)	.81
Carbohydrate intake (g/d)	402 (±125)	312 (±170)	.01
Fat intake (g/d)	92 (±42)	75 (±31)	.25

The genotype of rs9939609 polymorphism of the FTO gene was not significantly different between the two groups. Also, there was no significant difference between the two groups in terms of the dominant genetic model (AA and AT vs TT, *P* = .46). Two groups also did not differ significantly in terms of first menstrual age, menopausal age, smoking rate, alcohol consumption and physical activity.

The results of logistic regression on the association between BC and rs9939609 polymorphism also identified that there was no significant association between the risk of BC and FTO genotype (*P* = .46). Adjusting the effect of variables including age, family history of BC, abortion, BMI and number of pregnancies had no significant effect on the results (*P* = .5). Further adjustments with the variables of smoking, alcohol consumption, calorie and macronutrients intakes and physical activity did not have a significant effect on the results (Table 2).

**TABLE 2:** Logistic regression of the association between risk allele of rs9939609 FTO gene polymorphism and BC in all participants

Model	SE	OR	*P*
Model 1	0.25	1.21	.46
Model 2	0.31	1.24	.50
Model 3	0.54	2.05	.18

Note

Model 1: crude. Model 2: adjusted for age, family history of BC, postmenopausal status, months of breastfeeding, number of abortion, first menstruation age and the number of pregnancy. Model 3: further adjustments for smoking, alcohol, calorie and macronutrient intakes and physical activity.

In the next step, in order to investigate the effects of body mass index on the relationship between FTO gene polymorphism and BC, the analysis was limited to overweight people in two groups. Overweight individuals in the case group had significantly higher age, family history of BC, physical activity, calorie intake and carbohydrate intake than the control group (all *P* < .01), while the height and lactation duration of the case group were significantly lower than the control group (both *P* = .01).

Regarding the association between FTO gene and BC in overweight people, the frequency of rs9939609 polymorphism risk allele (allele A) in BC patients was significantly higher than the controls (*P* = .03, Table 3).

**TABLE 3:** Characteristics of overweight participants among the case and control groups

	Case (120)	Control (245)	*P*
Age (y)	59 (±8.7)	49 (±8.2)	.01
Height (cm)	156 (±5.3)	160 (±6)	.01
Weight (kg)	76.35 (±9.3)	77.77 (±9.1)	.40
BMI (kg/m^2^)	31.3 (±3.1)	30.3 (±3.5)	.08
Breastfeeding duration (mo)	37 (±28)	60 (±34)	.01
First menstruation age (y)	13.5 (±1)	13 (±1)	.70
Postmenopausal age (y)	45 (±5)	47 (±5)	.60
Family history of BC	46 (38%)	34 (14%)	.01
Number of pregnancies	3 (±1.8)	3 (±1.9)	.15
Smoking	4 (3%)	2 (1%)	.13
Using alcohol drinks	117 (97.1%)	242 (98.8%)	.45
Physical activity (h/d)	2.5 (±6.2)	1.5 (±1.4)	.01
FTO genotype for rs9939609 polymorphism			
TT	13 (10.5%)	86 (35%)	.16
AT	95 (79%)	132 (54%)	
AA	13 (10.5%)	27 (11%)	
Dominant model			
TT	13 (10.5%)	86 (35%)	.03
AA + AT	107 (89.5%)	159 (65%)	
Calorie intake (Kcal)	2847 (±1034)	2267 (±936)	.01
Protein intake (g/d)	92 (±48)	80 (±33)	.18
Carbohydrate intake (g/d)	409 (±152)	307 (±136)	.01
Fat intake (g/d)	99 (±50)	90 (±47)	.36

The results of logistic regression on the association between BC and FTO rs9939609 polymorphism in overweight people identified a significant difference between the case and control groups in terms of the frequency of allele risk of the FTO polymorphism (*P* = .04). Adjusting the effect of age, family history of BC, abortion, BMI and the number of pregnancies had no effect on the results (*P* = .04). However, after further adjustments for the lifestyle variables including smoking, alcohol consumption, macronutrients intakes and physical activity, this association was disappeared (Table 4). These results indicated that the FTO gene polymorphism may be associated with the risk of BC in overweight people and probably exert its effects through changes in lifestyle factors including diet, alcohol consumption and smoking.

**TABLE 4:** Logistic regression of the association between rs9939609 FTO gene polymorphism and BC in overweight participants

Model	SE	OR	*P*
Model 1	0.76	4.5	.04
Model 2	1.60	4.10	.04
Model 3	0.23	4.01	.98

Note

Model 1: crude. Model 2: adjusted for age, family history of BC, postmenopausal status, months of breastfeeding, number of abortion, first menstruation age and the number of pregnancy. Model 3: further adjustments for smoking, alcohol, calorie and macronutrient intakes and physical activity.

## 4 DISCUSSION

The present case‐control study found no significant association between BC and risk allele of FTO rs9939609 polymorphism in the participants with different BMI, in line with some previous studies.^20^, ^22^ For example, Mojaver et al found no significant association between rs9939609 FTO gene and the risk of BC among Iranian women.^21^ Also, Da Cunha et al found no association between this polymorphism and BC risk.^23^ On the other hand, Kaklamani et al and Zhao et al identified a significant association between several SNPs of the FTO gene including rs9939609 with the BC risk.^10^, ^25^ These differences in the obtained results might be related to different types of BC and various environmental, genetic and ethnic factors involved.^26^ Another important finding of this study was the significantly higher frequency of rs9939609 polymorphism risk allele in overweight persons with BC than in healthy persons with overweight. In line with this study, Mozafarizadeh et al reported that rs9939609 FTO gene polymorphism was significantly associated with the risk of BC in overweight persons.^26^ In addition, Kang et al reported that the status of BMI can influence on the association between rs9939609 polymorphism and BC.^27^ It is possible that the FTO genotype can influence the risk of BC only in overweight people. Interestingly, the association between FTO gene polymorphisms and breast cancer was reported to be influenced by the status of oestrogen receptors. Oestrogen may promote breast cancer cell proliferation through up‐regulation of FTO gene expression and activation of the PI3 K/Akt signalling pathway in oestrogen receptor‐positive patients.^28^

According to the present study, the association between FTO genotype and BC risk was disappeared after adjusting for lifestyle variables such as smoking, alcohol consumption, calorie and macronutrients intakes, and physical activity. So, it can be concluded that the FTO gene polymorphism is probably associated with BC in overweight persons and exerts its effects by modifying the lifestyle including dietary intake, alcohol consumption, or smoking. A review study by Doaei et al, which investigated the effect of diet on FTO gene expression in the hypothalamus, reported that the intake of macronutrients may be related to the expression level of FTO gene.^28^ Also, Park et al found that the amount of calorie intake from dietary fat and protein could be influenced by the FTO gene polymorphisms.^29^ It is plausible that the association between FTO gene and lifestyle is a mutual connection. The FTO gene polymorphisms can affect our food intake and physical activity. On the other hand, nutrient intake and physical activity may affect FTO gene expression level.^17^, ^18^, ^19^

Several studies have confirmed the association of alcohol consumption and smoking with the FTO gene and indicated that alcohol consumption and smoking are affected by the rs9939609 polymorphism of the FTO gene.^30^, ^31^ However, Hubacek et al found no association between this polymorphism and alcohol consumption. A recent study indicated that the effect of FTO polymorphisms on alcohol consumption may be altered under different environmental conditions.^32^

However, there were some limitations in our present study. First, different types of BC including the status of hormone receptors and also the stage of BC were not considered. Second, other anthropometric measurements such as persons' body fat were not assessed. Third, this study was limited to only one SNP of the FTO gene and other SNPs may have different associations with BC. Finally, the participant was not categorized based on their menopausal status and further studies are warranted.

## 5 CONCLUSION

In general, this case‐control study did not find any significant association between FTO gene polymorphism and BC. The FTO rs9939609 polymorphism risk allele was associated with the risk of BC in overweight people. However, adjustments for lifestyle factors including smoking, alcohol consumption, macronutrients intakes and physical activity disappeared the association. Further studies on the patients with different types of BC are needed to assess the possible effects of the FTO genotype on BC risk.

## CONFLICT OF INTEREST

The authors declare that they have no competing interests.

## AUTHOR CONTRIBUTION

**Saeid doaei:** Conceptualization (equal); Software (equal); Writing‐review & editing (equal). **Fatemeh Bourbour:** Methodology (equal); Validation (equal). **Samira Rastgoo:** Investigation (equal). **Mohammad Esmail Akbari:** Investigation (equal); Methodology (equal); Supervision (equal). **Maryam Gholamalizadeh:** Data curation (equal); Formal analysis (equal). **Azadeh Hajipour:** Data curation (equal); Validation (equal). **Alireza Moslem:** Visualization (equal); Writing‐original draft (equal). **Fereshteh Ghorat:** Investigation (equal). **Mostafa Badeli:** Resources (equal). **Seyedeh Elaheh Bagheri:** Validation (equal). **Atieh Alizadeh:** Methodology (equal); Writing‐review & editing (equal). **Zohreh Mokhtari:** Visualization (equal). **Samaneh Pishdad:** Writing‐original draft (equal). **Sepehr JavadiKooshesh:** Methodology (equal). **Ghasem Azizi Tabesh:** Investigation (equal). **Fatemeh Montazeri:** Visualization (equal). **Parvin Joola:** Methodology (equal); Validation (equal). **Shahla Rezaei:** Data curation (equal). **Masoomeh Dorosti:** Methodology (equal); Supervision (equal). **seyed Alireza Musavi Jarrahi:** Formal analysis (equal); Funding acquisition (equal).

## 6 ETHICS APPROVAL AND CONSENT TO PARTICIPATE

The study was approved by the ethics committee of Sabzevar University of Medical Sciences and Health Services (Reference Number: IR.MEDSAB.REC.1397.070), Khorasan Razavi, Iran. All participants involved provided written informed consent form before joining the project.

## Data Availability

The data sets used and/or analysed during the current study are available from the corresponding author on reasonable request.
